# Alpha_1_‐adrenergic stimulation selectively enhances endothelium‐mediated vasodilation in rat cremaster arteries

**DOI:** 10.14814/phy2.13703

**Published:** 2018-05-13

**Authors:** Ramesh C. Mishra, Mohammad M. Rahman, Michael J. Davis, Heike Wulff, Michael A. Hill, Andrew P. Braun

**Affiliations:** ^1^ Department of Physiology and Pharmacology Cumming School of Medicine University of Calgary Calgary Alberta Canada; ^2^ Dalton Cardiovascular Research Institute and Dept. of Medical Pharmacology and Physiology University of Missouri Columbia Missouri; ^3^ Department of Pharmacology University of California Davis Davis California

**Keywords:** Endothelium, eNOS, KCa channel, myogenic tone, vasodilation, *α*_1_‐Adrenoceptor

## Abstract

We have systematically investigated how vascular smooth muscle *α*_1_‐adrenoceptor activation impacts endothelium‐mediated vasodilation in isolated, myogenically active, rat cremaster muscle 1A arteries. Cannulated cremaster arteries were pressurized intraluminally to 70 mmHg to induce myogenic tone, and exposed to vasoactive agents via bath superfusion at 34°C. Smooth muscle membrane potential was measured via sharp microelectrode recordings in pressurized, myogenic arteries. The *α*_1_‐adrenergic agonist phenylephrine (25–100 nmol/L) produced further constriction of myogenic arteries, but did not alter the vasorelaxant responses to acetylcholine (0.3 *μ*mol/L), SKA‐31 (an activator of endothelial Ca^2+^‐dependent K^+^ channels) (3 *μ*mol/L) or sodium nitroprusside (10 *μ*mol/L). Exposure to 0.25–1 *μ*mol/L phenylephrine or 1 *μ*mol/L norepinephrine generated more robust constrictions, and also enhanced the vasodilations evoked by acetylcholine and SKA‐31, but not by sodium nitroprusside. In contrast, the thromboxane receptor agonist U46619 (250 nmol/L) dampened responses to all three vasodilators. Phenylephrine exposure depolarized myogenic arteries, and mimicking this effect with 4‐aminopyridine (1 mmol/L) was sufficient to augment the SKA‐31‐evoked vasodilation. Inhibition of L‐type Ca^2+^ channels by 1 *μ*mol/L nifedipine decreased myogenic tone, phenylephrine‐induced constriction and prevented *α*_1_‐adrenergic enhancement of endothelium‐evoked vasodilation; these latter deficits were overcome by exposure to 3 and 10 *μ*mol/L phenylephrine. Mechanistically, augmentation of ACh‐evoked dilation by phenylephrine was dampened by eNOS inhibition and abolished by blockade of endothelial KCa channels. Collectively, these data suggest that increasing *α*_1_‐adrenoceptor activation beyond a threshold level augments endothelium‐evoked vasodilation, likely by triggering transcellular signaling between smooth muscle and the endothelium. Physiologically, this negative feedback process may serve as a “brake” to limit the extent of vasoconstriction in the skeletal microcirculation evoked by the elevated sympathetic tone.

## Introduction

Changes in tissue blood flow are largely dependent upon the intraluminal diameter of small resistance arteries, which is jointly controlled by the myogenic reactivity of vascular smooth muscle, vasoactive molecules generated in the adjacent endothelium and the activity of sympathetic nerves innervating the arterial wall (Segal 2005; Bagher and Segal 2011; Westcott and Segal 2013). Each of these control processes depends upon diverse pathways and mechanisms at the cellular level, however, it remains poorly understood how these control processes interact with one another, as intraluminal diameter will ultimately be determined by the sum of their actions.

Several studies have provided compelling evidence that the vascular endothelium directly communicates with the adjacent smooth muscle via electrical and chemical signaling (e.g., myo‐endothelial coupling)(Busse et al. 2002; Edwards et al. 2010; Garland et al. 2011; Billaud et al. 2014), and related studies have demonstrated that signaling events originating in vascular smooth muscle can be detected in coupled endothelial cells (Dora et al. 1997; Yashiro and Duling 2000; Isakson et al. 2007; Nagaraja et al. 2012; Nausch et al. 2012; Garland et al. 2017). In particular, reports from Duling, Dora and coworkers have indicated that hormonal activation of vascular smooth muscle (VSM) *α*_1_‐adrenoceptors in resistance arteries can lead to elevated intracellular Ca^2+^ in the adjacent endothelial layer (Dora et al. 1997; Yashiro and Duling 2000; Isakson et al. 2007; Garland et al. 2017), which may subsequently impact endothelial function and regulation of vascular tone. In this study, we have hypothesized that concurrent *α*_1_‐adrenoceptor activation will either positively or negatively influence endothelium‐dependent vasodilation, and this impact will depend upon the degree of *α*_1_‐adrenoceptor activation (i.e., magnitude/direction of effect driven by the concentration of *α*_1_‐adrenoceptor agonist). This hypothesis was examined by investigating the influence of VSM *α*_1_‐adrenoceptor activation on the regulation of intraluminal diameter by endothelium‐dependent vasodilators in isolated, myogenically active rat skeletal muscle arteries. Experimentally, such qualitative effects would be observed as a quantitative enhancement or impairment of evoked, endothelium‐dependent vasodilation in the presence vs. the absence of *α*_1_‐adrenoceptor activation. Such *α*_1_‐mediated effects on endothelial function could arise from altered intracellular Ca^2+^ signaling (Dora et al. 1997; Yashiro and Duling 2000; Isakson et al. 2007), and/or responsiveness of VSM to vasoactive signals from the endothelium (e.g., changes in the sensitivity of VSM to endothelium‐derived signals).

The results of our study demonstrate the *α*_1_‐adrenergic agonists phenylephrine and noradrenaline, but not the thromboxane receptor agonist U46619, significantly enhanced endothelium‐dependent vasodilation in response to acetylcholine (ACh) and the K_Ca_ channel activator SKA‐31, whereas the vasodilatory response to sodium nitroprusside was unaffected in the presence of *α*_1_‐adrenoceptor activation. This observed enhancement of ACh‐ and SKA‐31‐mediated dilations by *α*_1_‐adrenoceptor agonist occurred in a concentration‐dependent manner, and required intact eNOS and/or endothelial K_Ca_ channel activities. Mechanistically, *α*_1_‐adrenoceptor evoked changes in vascular smooth muscle (VSM) Ca^2+^ levels, as revealed by L‐type Ca^2+^ channel inhibition with nifedipine. Increased membrane electrical resistance also appears to contribute to the positive effect of *α*_1_‐adrenergic signaling on ACh and SKA‐31‐mediated dilation. Collectively, these results provide new insights describing how graded activation of VSM *α*_1_‐adrenoceptors is able to modulate endothelium‐mediated vasodilation. Functionally, this intercellular communication may underlie a novel negative feedback mechanism in skeletal muscle resistance arteries to limit the active decrease in intraluminal diameter driven by *α*_1_‐adrenoceptor signaling and/or myogenic contraction.

## Methods and Materials

### Isolated vessel preparation

The experimental protocols used in this study were approved by the University of Calgary Animal Care Committee, and conform to the guidelines for the care and use of laboratory animals established by the Canadian Council on Animal Care and the Guide for the Care and Use of Laboratory Animals (NIH, 8th edition, 2011). Male Sprague‐Dawley rats (225–250 g body weight) were obtained from Charles River Laboratories and housed under standard conditions (i.e., 12 h day/light cycle, 22°C) with continuous access to food and water. Rats were injected intraperioneally with sodium pentobarbital (50 mg/kg) to induce surgical anesthesia (i.e., stage 3, loss of blink reflex), and cremaster muscles were then surgically removed and placed in a cooled (4°C) dissection chamber containing Krebs’ buffer (115 NaCl, 5 mmol/L KCl, 25 mmol/L NaHCO_3_, 1.2 mmol/L MgCl_2_, 2.5 mmol/L CaCl_2_, 1.2 mmol/L KH_2_PO_4_ and 10 mmol/L d‐glucose); pH was adjusted to 7.4 with 1 N NaOH (Meininger et al. 1991; Sheng et al. 2009). Euthanasia was completed by an overdose administration of sodium pentobarbital (150 mg/kg, I.P.). Following isolation, cremaster 1A arteries were cannulated on glass pipettes fitted in a pressure myography chamber (Living Systems, Burlington, VT) and the vessel lumen was filled with Krebs’ buffer containing 1% bovine serum albumin, pH was adjusted to 7.4. The cannulated vessel/chamber apparatus was placed on the stage of an inverted microscope, and one cannula end was closed and the other connected to a hydrostatic pressure column. The vessel was superfused with Krebs’ buffer at a constant flow of 7 mL/min, using a peristaltic pump and suction line. Bath solution was gassed with 95% air/5% CO_2_ and maintained at 34°C. The intraluminal pressure of cannulated vessels was increased in a step‐wise manner under no‐flow conditions and then maintained at 70 mmHg; vessels typically developed myogenic tone within 15–20 min. Intraluminal vessel diameter was continuously tracked using a video camera‐based imaging system (IonOptix, Milton, MA). Drugs were added to the bath via the peristaltic pump. Note that changing the experimental order in which vasodilators were applied to arteries did not change the magnitude of observed responses to a given dilator.

Drug‐induced changes in arterial intraluminal diameter (i.e., inhibition of myogenic tone, expressed in microns) were calculated as the difference in steady‐state diameter in the presence of a given vasodilator (D_drug_) and the intraluminal diameter under baseline conditions (D_basal_) measured immediately prior to a given drug exposure. Thus,

$$\text{Inhibition of Myogenic Contraction}\left(\mu m\right)=D_{\mathrm{drug}}-D_{\mathrm{basal}},$$

where D_drug_ = intraluminal diameter in the presence of a given vasodilator (e.g. ACh or SNP), D_basal_ = intraluminal diameter under basal myogenic tone at 70 mmHg. When vasodilatory responses were measured in the presence of myogenic tone + a constrictor agent (i.e. PE, NE or U46619), the D_basal_ value was calculated at the new baseline diameter observed in the presence of the constrictor, immediately prior to each vasodilatory response.

### Membrane Potential Recordings

Sharp microelectrodes were fabricated from borosilicate capillaries containing an internal filament (1.0 mm OD, 0.58 mm ID) using a Sutter P‐97 electrode puller. Microelectrodes were filled with 1 mol/L KCl and had tip resistances of 100–150 MΩ when placed in the bath solution. Voltage offsets for the microelectrodes were zeroed using the microelectrode amplifier (NPI model SEC‐05X). Isolated cremaster 1A arteries were cannulated and pressurized to 60 mmHg, as described above. Following the development of myogenic tone, a microelectrode was impaled into the vessel wall and smooth muscle membrane potential (*V*_m_) was monitored continuously and recorded on a computer using LabView‐based acquisition software. A cell impalement was considered to be successful if a sharp negative deflection in *V*_m_ was observed upon insertion of the microelectrode into the outer smooth muscle layer and a stable recording of Vm was obtained for >1 min. Once a baseline *V*_m_ was established, the amplifier was switched to current clamp mode and three negative current pulses of increasing magnitude (range, −30 to −240 pA, 2 sec duration) were injected. Corresponding deflections in *V*_m_ from the baseline were recorded and the amplitudes were plotted vs. injected current step size. The calculated slope of the relation (i.e., *V*_m_ vs. current step size) yielded the electrical resistance of the smooth muscle membrane (*R*_m_). Following measurements of *V*_m_ and *R*_m_ under control conditions, the bath solution was switched to one containing 1 *μ*mol/L phenylephrine, which caused the artery to constrict. In some instances, microelectrode impalements were maintained and continuous recordings of *V*_m_ were obtained; however, the PE‐induced constriction typically led to loss of the impalement, necessitating the reinsertion of the microelectrode. Once a stable basal Vm was achieved in the presence of PE, current injections were repeated and Rm calculated as described above. In a few instances, measurements of smooth muscle *V*_m_ and *R*_m_ were obtained following PE washout and recovery of the artery to basal myogenic conditions.

### Reagents

Acetylcholine chloride, EGTA (ethylene glycol‐*bis*(2‐aminoethylether)‐*N*,*N*,*N*’,*N*’‐tetraacetic acid), Phenylephrine hydrochloride ((*R*)‐(‐)‐1‐(3‐hydroxyphenyl)‐2‐methylaminoethanol hydrochloride), (‐)Norepinephrine bitartrate salt, 4‐AP (4‐aminopyridine), SNP (sodium nitroprusside), DMSO (dimethyl sulfoxide), Nifedipine (1,4‐dihydro‐2,6‐dimethyl‐4‐(2‐nitrophenyl)‐3,5‐pyridinedicarboxylic acid dimethyl ester), Pinacidil (***N*** **‐cyano‐N′‐4‐pyridinyl‐** ***N*** **″‐(1,2,2‐trimethylpropyl)guanidine monohydrate**), U46619 (5*Z*)‐7‐[(1*R*,4*S*,5*S*,6*R*)‐6‐[(1*E*,3*S*)‐3‐hydroxy‐1‐octenyl]‐2‐oxabicyclo[2.2.1]hept‐5‐yl]‐5‐heptenoic acid, and all required chemicals to prepare physiological solutions were purchased from Sigma‐Aldrich (Oakville, ON, Canada). Euthanyl (sodium pentobarbital, 250 mg/mL) was purchased from Bimeda‐MTC Animal Health Inc, Cambridge, ON, Canada. SKA‐31 (naphtho [1, 2‐*d*] thiazole‐2‐ylamine) was synthesized as previously described (Sankaranarayanan et al. 2009). SKA‐31, pinacidil and nifedipine were prepared as 10 mmol/L stock solutions in DMSO and then diluted directly into the external bath solution. The final concentration of DMSO reaching the tissue was typically 0.02% (vol/vol) or less. In preliminary experiments, we observed that a considerably higher concentration of DMSO (0.2% v/v final) had no effect on either basal myogenic tone or the responsiveness of cremaster arteries to vasodilatory agents (*n* = 2, data not shown).

### Statistical analysis

**Figure 1:**
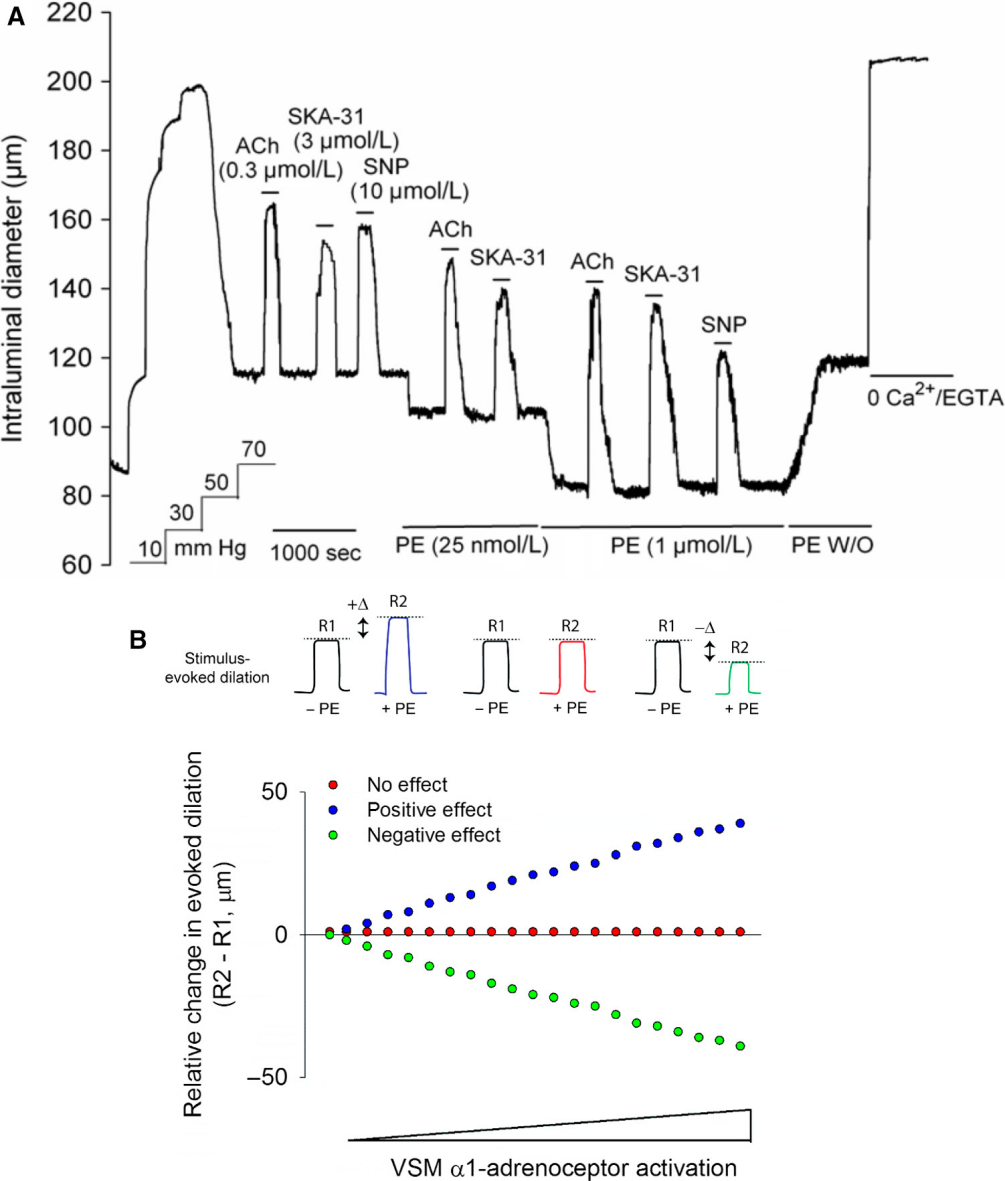
Panel (A) shows a representative tracing of stimulus‐evoked dilations in a single cannulated cremaster artery in the absence and presence of phenylephrine (PE). Addition of acetylcholine (ACh, 0.3 *μ*mol/L), SKA‐31 (3 *μ*mol/L), or SNP (10 *μ*mol/L) produced rapid and reversible increases in intraluminal diameter, as indicated by horizontal bars above the tracing. Exposures to vasodilatory agents were then repeated in the presence of either 25 nmol/L PE or 1 *μ*mol/L PE, followed by washout (W/O) of PE from the bath, as shown by the horizontal bars below the tracing. Superfusion with a nominally Ca^2+^‐free Krebs’ buffer containing 2 mmol/L EGTA was performed to obtain the maximal passive intraluminal diameter at 70 mmHg. The tracing is representative of 6 cremaster arteries subjected to the same experimental protocol. The schematic in panel (B) depicts the hypothetical influence of vascular smooth muscle *α*_1_‐adrenoceptor activation on stimulus‐evoked dilation of a myogenically active skeletal muscle artery. The idealized tracings in the upper panel depict reversible, stimulus‐induced vasodilatory responses (i.e. change in intraluminal diameter from the basal level) in a myogenic artery and how the amplitude of these primary responses may be hypothetically altered in the presence of an *α*_1_‐adrenoceptor agonist (e.g. PE). The pair of tracings on the left illustrates a positive influence of PE on the amplitude of evoked dilation (R2 response, blue trace) compared with the control (R1, black trace), as indicated by the larger vasodilation (i.e., R2 > R1). The right‐hand tracings show a negative effect of PE on the induced vasodilatory response (i.e., R2 < R1, green trace), while the middle tracings demonstrate no change in the amplitude of evoked dilation in the presence of PE (R2 = R1, red trace). A plot of the **relative** changes in the amplitude of evoked dilation ± PE (i.e. R2–R1) illustrates these three possible influences of PE (i.e., positive effect, blue symbols; no effect, red symbols; negative effect, green symbols) and how they may change with increasing VSM *α*_1_‐adrenoceptor activation.

Data are presented as mean ± SEM for *n* arteries obtained from *N* different animals. Often, only one artery was examined per animal, due to the lengthy duration of the experimental protocol. For each artery, functional responses to pharmacologic agents were first obtained under control conditions and then in the presence of a stated treatment (e.g., 1 *μ*mol/L phenylephrine exposure). Each vessel thus served as its own control for a given manipulation. The difference in the magnitude of response to a given agent under control and treatment conditions (i.e., R2 ‐ R1) was then calculated and presented graphically, as described in Figure 1B. In some cases, a calculated set of differences was analyzed using a one sample *t*‐test (SigmaPlot ver12) to determine if the mean was statistically different than zero (see Figs. 3, 6B and 8C). For other data, a statistically significant difference for the same stimulus under two experimental conditions was evaluated, using either a Student's unpaired *t*‐test (see Fig. 8B) or a one‐way ANOVA, followed by a Tukey post hoc test. A calculated *P* < 0.05 was taken to signify statistical significance.

## Results

### The *α*_1_‐adrenoceptor agonist phenylephrine selectively enhances endothelium‐dependent responses in myogenically active rat cremaster arteries

To examine the interaction between endothelium‐dependent regulation of intraluminal diameter in a small resistance artery in the absence and presence of *α*_1_‐adrenoceptor stimulation, we utilized cannulated, myogenically active rat cremaster arteries. As displayed in Figure 1A, brief exposure to the endothelium‐dependent dilator acetylcholine (ACh), at a concentration (0.3 *μ*mol/L) predicted to produce ~60% dilation(Sheng et al. 2009), reversibly inhibited myogenic tone at 70 mmHg, leading to an increase in intraluminal diameter of 55.8 ± 6.6 *μ*m (*n* = 67 arteries). A similar result was observed for 3 *μ*mol/L SKA‐31, a small molecule activator of endothelial small and intermediate‐conductance, Ca^2+^‐activated K^+^ channels (K_Ca_ 2.3 and K_Ca_ 3.1, respectively) (change in intraluminal diameter from baseline = 47.2 ± 5.8 *μ*m, *n* = 67). The concentration of SKA‐31 used experimentally approximated its EC_50_ value in rat cremaster arteries (Mishra et al. 2015). Note that the SKA‐31 solvent (i.e. DMSO) alone had no effects on baseline myogenic tone at bath concentrations ≥ 10‐fold higher than normally used (data not shown). The nitrovasodilator sodium nitroprusside (SNP), which acts directly on VSM to inhibit myogenic tone(Rubanyi and Vanhoutte 1985; Brizzolara and Burnstock 1991; Mishra et al. 2015), was used as an internal control and increased intraluminal diameter by 46.8 ± 4.9 *μ*m (*n* = 67). The experimentally determined EC_50_ value for SNP‐evoked vasodilation in our vessels was 6.0 *μ*mol/L (data not shown). When normalized to the total amount of active myogenic constriction generated in these arteries (85.6 ± 8.7 *μ*m, *n* = 67), the percentage inhibition of myogenic tone evoked by ACh, SKA‐31 and SNP was calculated to be 65.2%, 55.1% and 54.7%, respectively. The procedure for this latter calculation is described in detail elsewhere (Mishra et al. 2015).

In the presence of a low concentration of the *α*_1_‐adrenergic agonist phenylephrine (PE, 25 nmol/L), which itself caused a further reduction in intraluminal diameter, dilations in response to ACh, SKA‐31 and SNP were largely unchanged. However, when the same artery was exposed to a higher concentration of PE (1 *μ*mol/L), intraluminal diameter decreased further and ACh and SKA‐31 now evoked markedly larger dilations compared with those observed in the presence of 25 nmol/L PE. In contrast, 1 *μ*mol/L PE exposure did not change the dilatory response to SNP. Control experiments further demonstrated that repeated exposures to ACh, SKA‐31 and SNP (3 for each agent) produced stable vasodilatory responses over a period of ~4 h, in which exposures to a given agent were separated by ~60 min (data not shown). Washout of PE from the bath always resulted in return of intraluminal diameter to a level approximating the initial baseline value, strongly suggesting that myogenically active arteries retained their functional integrity and responsiveness throughout the protocol.

### Systematic analysis of *α*_1_‐adrenergic effects on evoked vasodilation by endothelium‐dependent and ‐independent agents

The upper panel in Figure 1B depicts the possible alterations in endothelium‐dependent vasodilation in the presence of VSM *α*_1_‐adrenoceptor activation (i.e., augmented, impaired or unchanged), as identified by the measurable differences in dilatory responses (i.e., R2–R1) in the presence and absence of an *α*_1_‐adrenergic agonist (e.g., phenylephrine, PE). The plot in Figure 1B further illustrates how these quantified differences in vasodilatory responses may change with increasing concentrations of the *α*_1_‐adrenergic agonist (for simplicity, only monophasic relations are shown). Revisiting the data presented in Figure 1A, two points are immediately evident: 1) *α*_1_‐adrenoceptor activation promotes an augmentation of endothelium‐evoked dilation (i.e. R2–R1), and 2) this *α*_1_‐adrenergic facilitation of evoked dilation occurs in a concentration‐dependent manner. We further speculate that these observed phenomena may follow the prediction described by the plotted blue symbols in Figure 1B.

**Figure 2:**
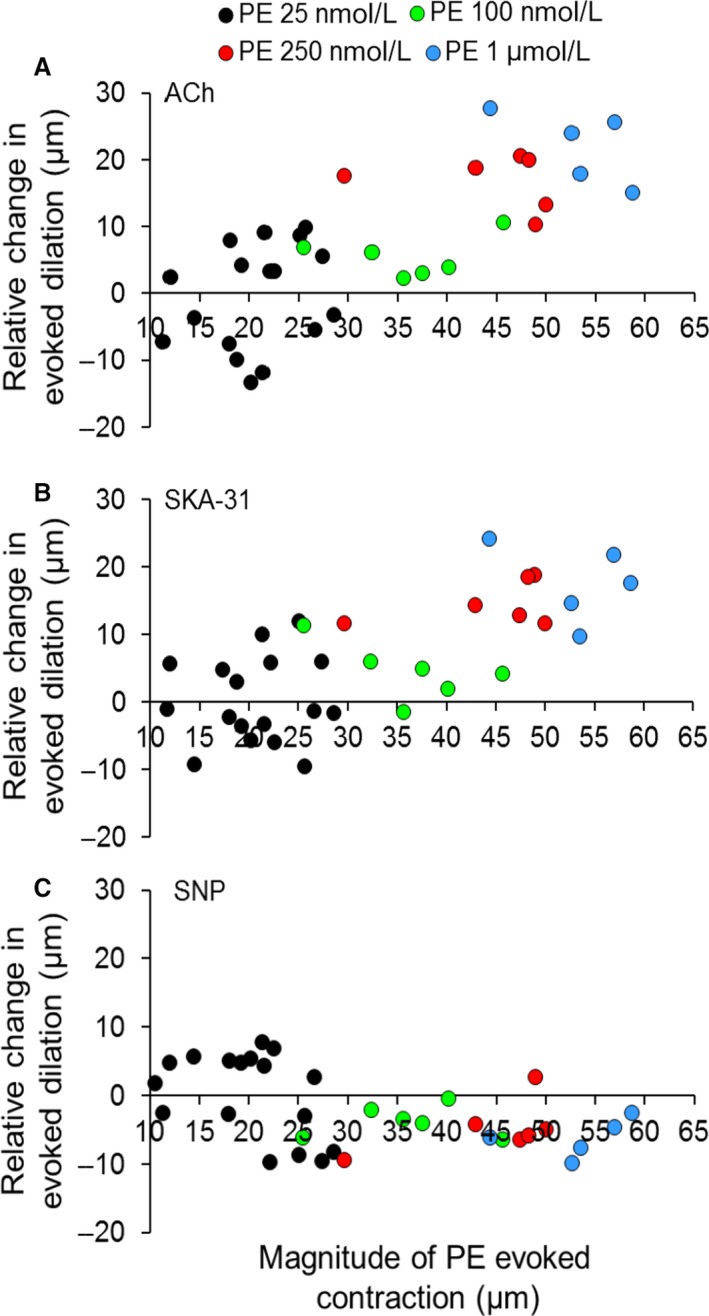
Quantification of drug‐induced changes in intraluminal diameter in myogenically active cremaster arteries in the absence and presence of increasing concentrations of phenylephrine (PE). For the scatter plots shown in panels A, B, and C, each symbol depicts the relative change, or calculated difference in the magnitude of dilatory responses (i.e., R2–R1) evoked by a given vasodilator in the absence (R1) or presence of the indicated concentration of PE (R2), as denoted by the symbol legend at the top of the figure. Note that each plotted symbol represents an individual artery. The position of a symbol along the x‐axis reflects the magnitude of PE‐induced contraction in microns (relative to the level of basal myogenic tone) observed for a given concentration of PE. Experimentally, control dilatory responses were first obtained following acute bath exposure to ACh (0.3 *μ*mol/L), SKA‐31 (3 *μ*mol/L), and SNP (10 *μ*mol/L) and these responses were then repeated following bath addition of a fixed concentration of PE, as shown in Figure 1A. Arteries were typically exposed to only a single PE concentration during an experiment, followed by washout to ensure reversibility of PE‐induced contraction. Data were collected from 32‐34 arteries, which were isolated from a total of 34 animals.

The observed enhancement of endothelium‐dependent vasodilation by *α*_1_‐adrenoceptor signaling largely reproduces the basic observation described by Duling and colleagues(Dora et al. 1997, 2003; Yashiro and Duling 2000), and was investigated in a more extensive manner by examining the vasodilatory effects of the endothelium‐dependent dilators ACh (0.3 *μ*mol/L) and SKA‐31 (3 *μ*mol/L) and the smooth muscle agent SNP (10 *μ*mol/L) in the absence and presence of 25, 100, 250, and 1000 nmol/L PE. This range of PE concentrations would be expected to produce increasing activation of VSM *α*_1_‐adrenoceptors in cremaster arteries(Faber 1988; Jackson et al. 2008; Moore et al. 2010a), which would be reflected functionally by elevated levels of contraction (i.e., reductions in intraluminal diameter) in myogenically active arteries. Differences in the magnitude of stimulus‐evoked dilation could then be measured, using the strategy depicted in Figure 1B (i.e., R2–R1) and plotted versus the magnitude of PE‐induced contraction at the indicated concentration. Pharmacologically, PE‐stimulated contraction correlates with *α*_1_‐adrenoceptor activation/occupancy in VSM (Ruffolo 1987). Quantitatively, PE‐dependent decreases in intraluminal diameter at 70 mmHg ranged from 10 *μ*m at 25 nmol/L PE up to ~60 *μ*m at 1 *μ*mol/L PE. This analytical approach thus allowed us to evaluate how a relative change in stimulus‐evoked dilation (i.e., R2–R1) correlated with the magnitude of PE‐induced constriction (i.e., a reflection of VSM *α*_1_‐adrenoceptor activation). The scatter plot displayed in Figure 2A shows that ACh‐evoked dilation was clearly augmented in the presence of either 250 nmol/L or 1 *μ*mol/L PE (denoted by symbols appearing above the zero difference line), but not in the presence of either 25 or 100 nmol/L PE (i.e., symbols clustered near the zero difference line). Note that each symbol in the plot represents a measurement from an individual artery. A similar pattern of results was observed for SKA‐31‐induced dilations in the absence and presence of PE exposure (Fig. 2B). In contrast, SNP‐evoked dilations remained unchanged over the same range of PE concentrations (Fig. 2C), indicating that the observed PE‐mediated effect was selective for endothelium‐mediated vasodilatory responses.

**Figure 3:**
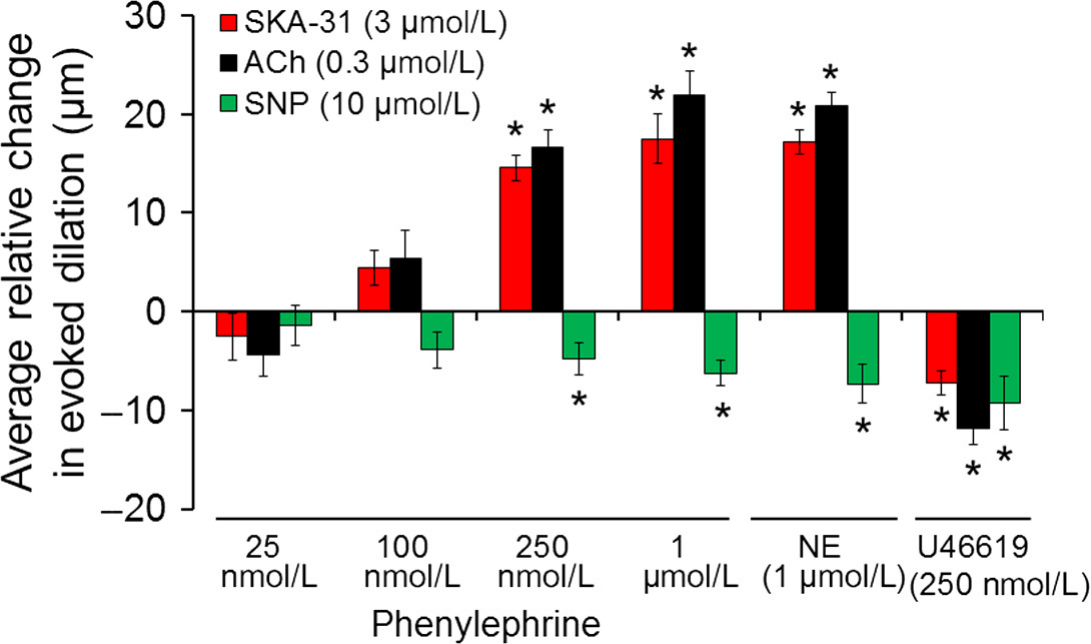
Quantification of the average relative changes in evoked dilation in the absence and presence of vasoconstrictor agonists. Average differences in the magnitude of evoked vasodilations by ACh, SKA‐31 and SNP (i.e., R2–R1) were calculated from observed responses in the absence and presence of the vasoconstrictors denoted beneath the bars. The asterisk (*) denotes a statistically significant difference in the mean calculated difference relative to zero, as determined by an unpaired Student's *t* test (*P* < 0.05). For PE‐associated data, average values were calculated from 32‐34 arteries; for NE‐associated data, 11 arteries were analyzed; for U46619‐associated data, 7 arteries were analyzed. Each artery was obtained from an individual animal.

For ACh, SKA‐31, and SNP, the relative differences in evoked dilation (i.e. R2–R1 data) were further used to calculate average values at each concentration of PE exposure. As presented in Figure 3, calculated mean differences in ACh responses in the presence of 25 and 100 nmol/L PE are −4.4 ± 2.1 *μ*m and +5.4 ± 2.8 *μ*m, respectively, and are not statistically different from zero. For SKA‐31 responses under the same PE conditions, average values are −2.5 ± 2.4 *μ*m and +4.4 ± 2.7 *μ*m, and are not different from zero. However, in the presence of either 250 nmol/L or 1 *μ*mol/L PE, calculated mean differences in ACh‐evoked dilations are +16.7 ± 1.7 *μ*m and +22.0 ± 2.4 *μ*m, respectively. In the case of SKA‐31, average differences (i.e., R2–R1) calculated in the presence and absence of 250 nmol/L and 1 *μ*mol/L PE are +14.5 ± 1.3 *μ*m and +17.5 ± 2.6 *μ*m, respectively. Thus, ACh and SKA‐31 evoked statistically greater dilations in the presence of 250 nmol/L and 1 *μ*mol/L PE, compared with responses in the absence of PE (*P* < 0.05). Collectively, these data demonstrate that VSM *α*_1_‐adrenoceptor activation functionally augments endothelium‐dependent inhibition of myogenic tone in a concentration‐dependent manner in rat skeletal muscle arteries.

Similar to ACh and SKA‐31, vasodilatory responses evoked by 10 *μ*mol/L SNP were unchanged in the presence of bath‐applied PE (25 nmol/L and 100 nmol/L), as evidenced by the scatter of individual calculated differences around the line indicating zero net effect (Fig. 2C). In the presence of either 250 nmol/L or 1 *μ*mol/L PE, however, the average SNP‐evoked vasodilations were modestly, but significantly less than the control responses (−4.8 ± 1.7 *μ*m and −6.2 ± 2.3 *μ*m, respectively) (Fig. 3). Taken together, these observations indicate that VSM *α*_1_‐adrenoceptor activation selectively enhances vasodilatory responses to the endothelium‐dependent agents ACh and SKA‐31 at higher agonist concentrations, and slightly depresses SNP‐evoked dilation.

### The endogenous *α*‐adrenoceptor agonist norepinephrine augments vasodilatory responses by endothelium‐dependent agents

**Figure 4:**
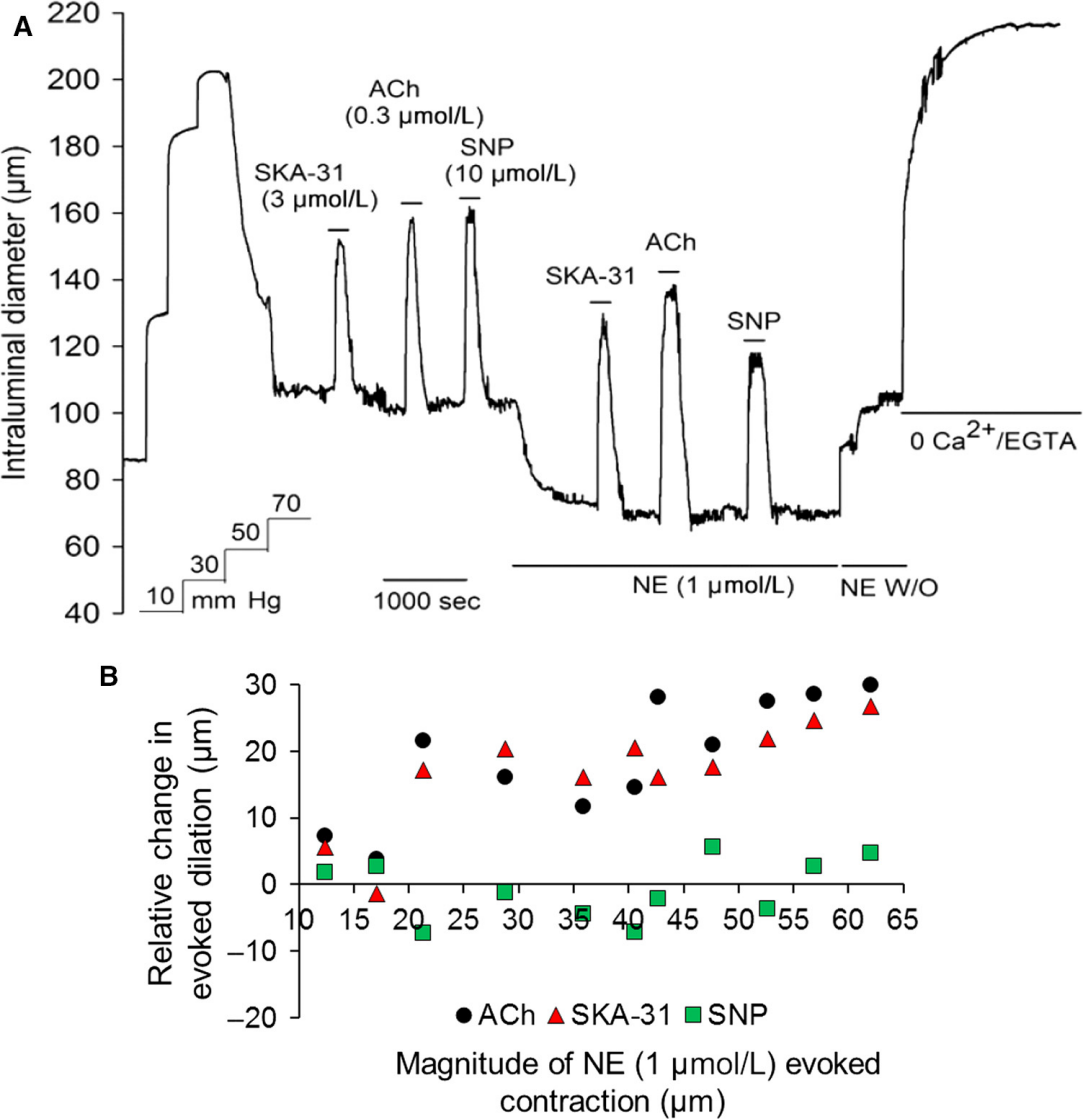
Norepinephrine (NE, 1 *μ*mol/L) enhanced responses to ACh and SKA‐31, but not SNP in rat cremaster artery. Panel (A) shows a representative tracing of vasodilatory responses evoked by ACh, SKA‐31 and SNP in a myogenic artery in the absence and presence of 1 *μ*mol/L NE, as indicated by the horizontal bars above and below the tracing. Bath exposure to 0 Ca^2+^ Krebs’ solution + 2 mmol/L EGTA produced maximal passive diameter of the artery at 70 mmHg. The scatter plot in panel B quantifies the relative change, or calculated difference in the magnitude of dilatory responses (i.e., R2–R1) evoked by a given vasodilator in the presence (R2) or absence of 1 *μ*mol/L NE (R1). Individual symbols represent individual responses to the noted vasodilator, as defined by the legend. The position of a symbol along the x‐axis reflects the magnitude of NE‐induced contraction in microns (relative to the level of basal myogenic tone). Data were collected from a total of 11 animals.

As depicted in Figure 4, activation of VSM *α*‐adrenoceptors by norepinephrine (NE, 1 *μ*mol/L) produced a similar degree of vasoconstriction (37.6 ± 7.8 *μ*m) in myogenically active, cremaster arteries as that seen with 0.25–1 *μ*mol/L PE (Fig. 2). Exposure to NE further augmented the vasodilatory responses to the endothelium‐dependent agents ACh and SKA‐31, but not to the nitrovasodilator SNP, as illustrated by the scatter plot shown in Figure 4B. Calculated mean differences in dilatory responses evoked by 0.3 *μ*mol/L ACh, 3 *μ*mol/L SKA‐31 and 10 *μ*mol/L SNP in the presence and absence of 1 *μ*mol/L NE are +20.8 ± 1.9 *μ*m, +17.2 ± 2.0 *μ*m and −7.3 ± 2.5 *μ*m, respectively (values also displayed in Fig. 3).

### Stimulus‐evoked vasodilatory responses are not enhanced by the thromboxane A_2_ receptor (TP) agonist U46619

**Figure 5:**
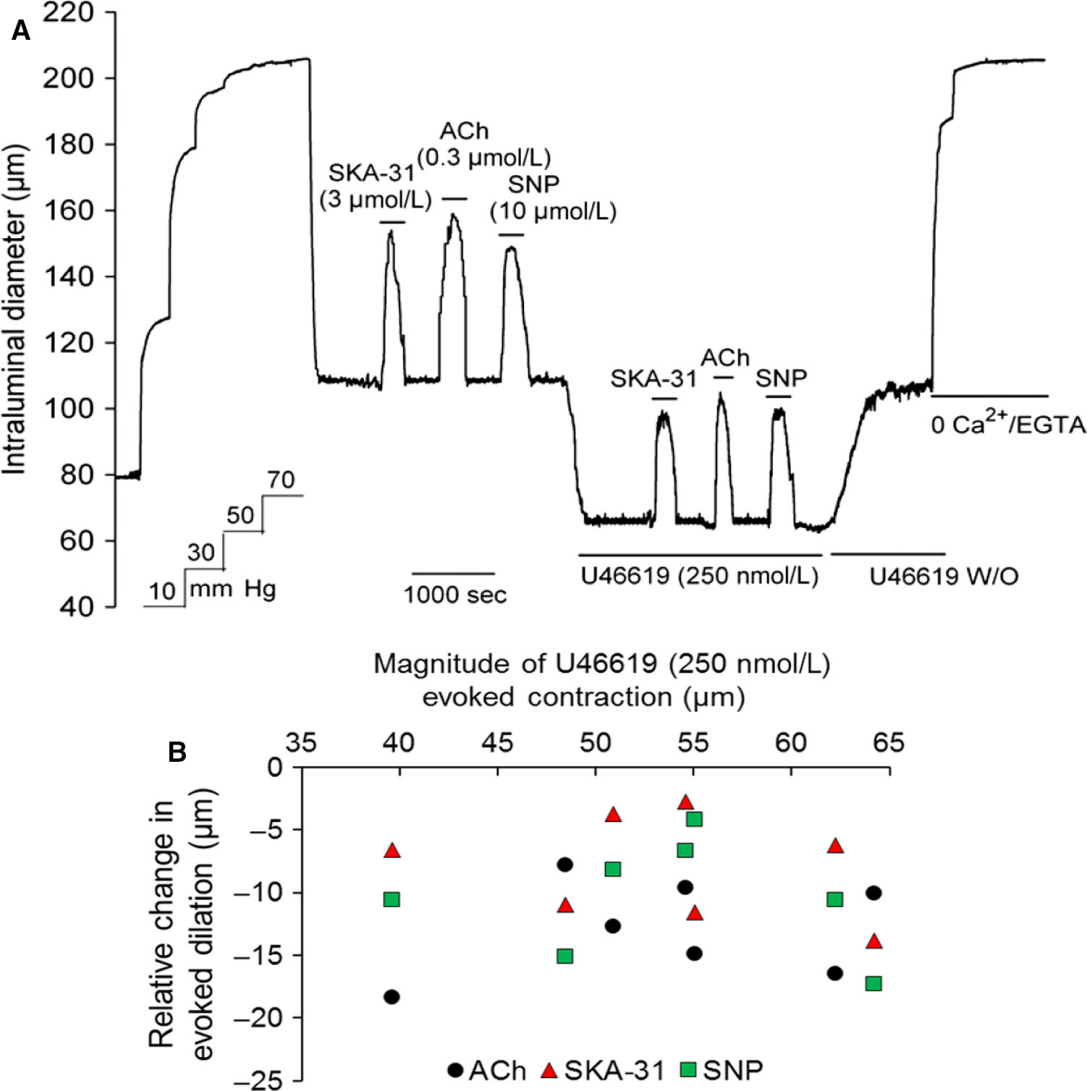
The thromboxane agonist U46619 (250 nmol/L) impairs vasodilatory responses to ACh, SKA‐31 and SNP in rat cremaster artery. Panel A shows a representative tracing of vasodilatory responses evoked by ACh, SKA‐31 and SNP in a myogenic cremaster artery in the absence and presence of 250 nmol/L U46619, as denoted by the horizontal bars above and below the representative tracing. Following washout (W/O) of U46619, maximal passive diameter at 70 mm Hg was determined in the presence of 0 Ca^2+^ Krebs’ solution containing 2 mmol/L EGTA. The scatter plot in panel B depicts the relative changes in drug‐induced vasodilatory responses for ACh, SKA‐31 and SNP in the presence and absence of 250 nmol/L U46619 (i.e., R2–R1). The position of a symbol along the x‐axis reflects the magnitude of U46619‐induced contraction in microns (relative to the level of basal myogenic tone). Data were collected from a total of 7 animals.

To determine if the observed endothelium‐dependent vasodilatory responses could also be augmented by a different vasoconstrictor pathway, we investigated the effects of TP receptor activation by U46619. In contrast to the facilitation observed with the *α*‐adrenergic agonists PE and NE (Figs. 2, 3 and 4), evoked dilations were significantly reduced for ACh (−11.8 ± 1.7 *μ*m) and SKA‐31 (−7.2 ± 1.2 *μ*m) and the NO donor SNP (−9.3 ± 2.7 *μ*m) following activation of VSM TP receptors with U46619, compared with control responses (Figs. 5 and 3). The magnitude of vasoconstriction induced by 250 nmol/L U46619 in myogenically active cremaster arteries (51.8 ± 5.1 *μ*m, *n* = 7) represented ~80% of the maximal U46619‐induced contraction (data not shown) and was comparable to the decreases in intraluminal diameter produced by 1 *μ*mol/L PE and 1 *μ*mol/L NE. These observations indicate that a difference in the extent of vasoconstrictor‐induced tone per se cannot account for the observed enhancement of endothelium‐evoked dilations by PE and NE, and impairment by U46619.

### The voltage‐gated K^+^ channel blocker 4‐aminopyridine selectively augments stimulus‐evoked dilations

**Figure 6:**
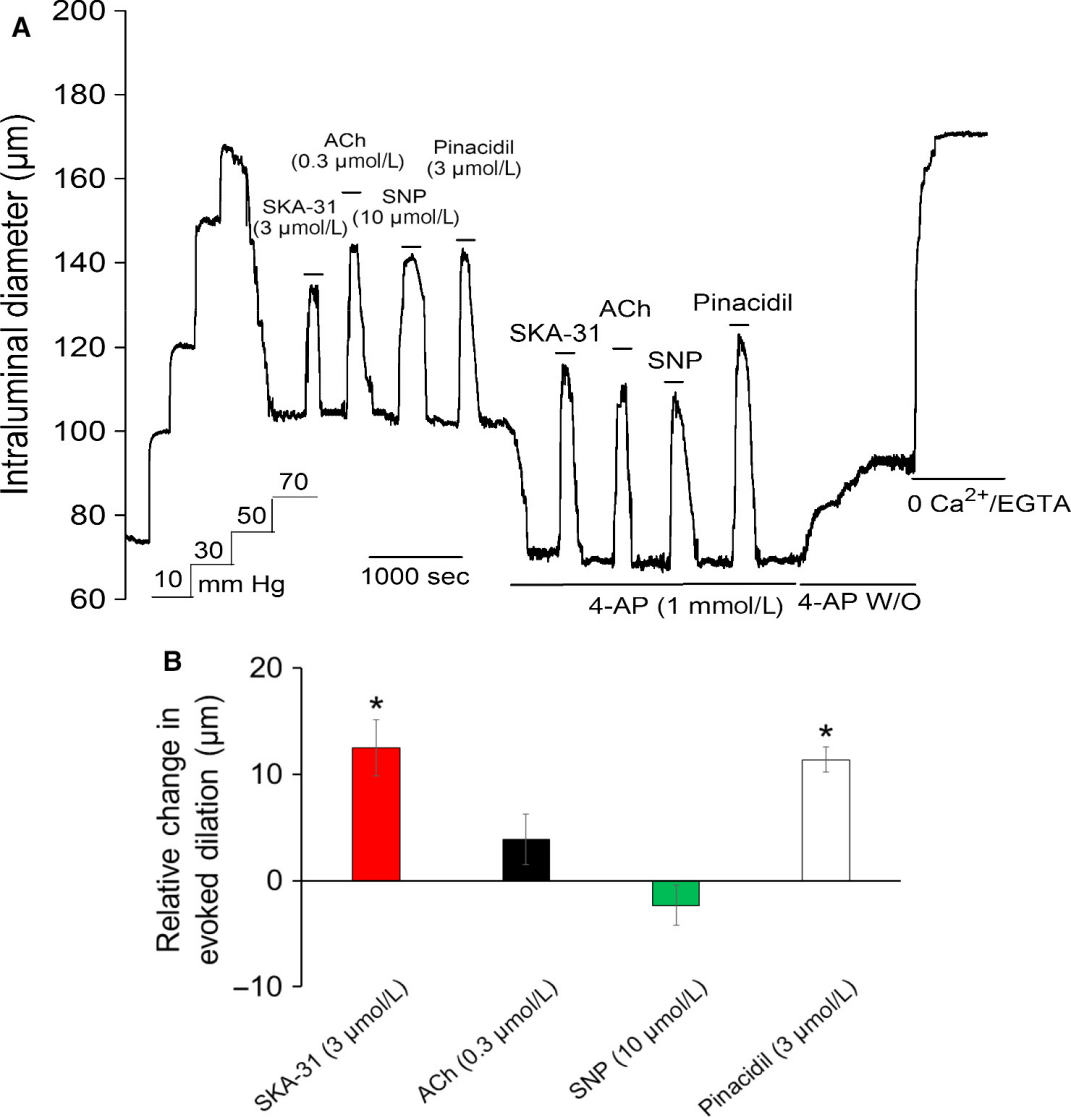
The voltage‐gated K^+^ channel blocker 4‐aminopyridine (4‐AP) augments vasodilatory responses to SKA‐31 and pinacidil, activators of endothelial and smooth muscle K^+^ channels, respectively. The representative tracing in panel (A) depicts vasodilatory responses to ACh (0.3 *μ*mol/L), SKA‐31 (3 *μ*mol/L), SNP (10 *μ*mol/L) and the K‐ATP channel activator pinacidil (3 *μ*mol/L) in both the absence and presence of 1 mmol/L 4‐AP, as denoted by the horizontal bars above and below the tracing. Following washout (W/O) of 4‐AP, maximal passive diameter of the artery was determined in the presence of 0 Ca^2+^ Krebs’ solution + 2 mmol/L EGTA. The histogram in panel B quantifies the average relative changes in stimulus‐evoked dilation (i.e., R2–R1) in the presence (R2) and absence (R1) of 4‐AP. Data are presented as mean ± SEM for 7 cremaster arteries. The asterisk (*) denotes a statistically significant difference in the magnitude of evoked dilation relative to the control condition, as determined by an unpaired Student's *t* test (*P* < 0.05).

*α*1‐Adrenergic stimulation leads to VSM membrane depolarization, which contributes to the contractile response by directly promoting voltage‐dependent, external Ca^2+^ entry and elevation of intracellular‐free Ca^2+^ (Miriel et al. 1999; Mistry and Garland 1999; Zou et al. 2000; Potocnik and Hill 2001; Li and Janssen 2002; Tran et al. 2012). We thus hypothesized that membrane depolarization alone may be sufficient to promote enhancement of endothelium‐dependent vasodilation by ACh and SKA‐31. To examine this possibility, myogenically active arteries were exposed to 4‐aminopyridine (4‐AP, 1 mmol/L), a nonselective blocker of voltage‐gated K^+^ channels in VSM(Nelson and Quayle 1995). Functionally, bath application of 4‐AP caused a further decrease in arterial intraluminal diameter (37.3 ± 3.1 *μ*m, *n* = 7) that was consistent with a depolarization‐induced elevation of cytosolic Ca^2+^ in VSM, and comparable in magnitude to the constrictions produced by PE, NE and U46619 (Fig. 6). These findings thus agree with earlier data showing that 4‐AP exposure caused both a decrease in intraluminal diameter and a membrane depolarization in myogenic cerebral arteries from rabbit (Knot and Nelson 1995). Functionally, 4‐AP significantly augmented the vasodilatory response to SKA‐31, an activator of endothelial K_Ca_ channels(Sankaranarayanan et al. 2009; Wulff and Köhler 2013), but surprisingly, did not produce a significant enhancement of the ACh response (Fig. 6B). Under the same conditions, 4‐AP also augmented the dilatory response to the smooth muscle K‐ATP channel activator pinacidil, which directly relaxes VSM via membrane hyperpolarization, and a decrease in L‐type Ca^2+^ channel activity (Nelson and Quayle 1995; Quayle et al. 1997). Mechanistically, the hyperpolarizing currents (I) generated in response to either SKA‐31 or pinacidil would drive greater VSM hyperpolarization, due to the increase in VSM membrane resistance (*R*_m_) induced by 4‐AP, as explained by Ohm's Law (*V*_m_ = I × *R*_m_), leading to augmented relaxation.

### *α*_1_‐Adrenoceptor activation depolarizes VSM membrane potential and increases membrane resistance

**Figure 7:**
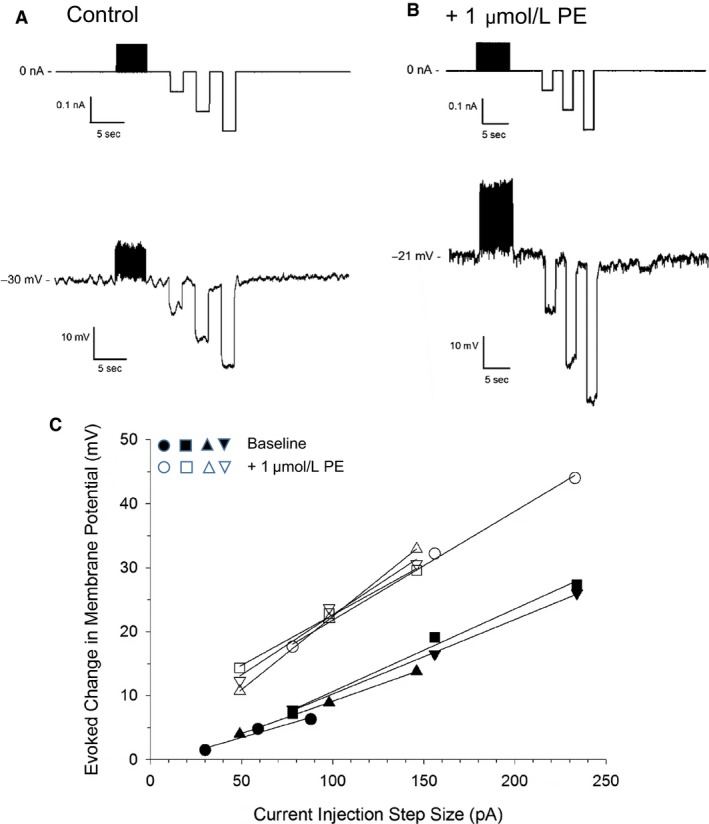
Effects of phenylephrine (PE) on VSM membrane resistance in cannulated, myogenically active cremaster arteries. Following establishment of myogenic tone, a microelectrode was stably impaled in the arterial wall and basal membrane potential (*V*_m_) was recorded. Three negative, step‐wise current injections of increasing magnitude were then applied, as shown by the upper tracing in panel (A). The lower panel shows the membrane voltage deflections recorded in response to these 2‐sec current injections. Panel (B) illustrates the membrane voltage changes in response to the same series of current injections in the same artery following exposure to 1 *μ*mol/L PE. These tracings are representative of 4 experiments performed in 3 separate arteries. The graph in panel (C) plots the magnitude of membrane voltage deflections versus the step size of corresponding current injections. Closed symbols represent *V*_m_ deflections observed under basal conditions; open symbols illustrate measurements made in the presence of 1 *μ*mol/L PE. Straight lines represent a linear regression fit to each set of data points and the slopes of the lines were used to calculate VSM membrane resistance in MΩ.

Observations in the presence of the *K*_v_ channel blocker 4‐AP prompted us to examine the extent to which *α*_1_‐adrenoceptor activation induced membrane depolarization in cannulated, myogenically active cremaster arteries. To do so, we performed single microelectrode measurements of VSM membrane potential (*E*_m_) ± *α*_1_‐adrenergic stimulation. In the absence of PE, the steady‐state Em of myogenic arteries pressurized to 60 mmHg was found to be −33.8 ± 3.2 mV, and addition of 1 *μ*mol/L PE to the impaled artery led to a further depolarization of *E*_m_ to −23.5 ± 1.2 mV (*P* < 0.05 compared with control *E*_m_, *n* = 4–5 recordings). To determine if the PE‐induced Em depolarization resulted from a decrease in membrane resistance (*R*_m_), due to the opening of cation‐selective ion channels in VSM, or an increase in resistance indicative of VSM K^+^ channel inhibition, we injected hyperpolarizing current pulses (range = 30–240 pA) via the microelectrode to elicit membrane voltage deflections (Fig. 7A and B). By plotting the magnitude of evoked voltage deflections versus the size of injected current steps and calculating *R*_m_ from the slopes of the linear relations (i.e., R = V/I), we observed that basal VSM membrane resistance significantly increased from 107.3 ± 20.0 MΩ to 186.2 ± 31.2 MΩ in the presence of 1 *μ*mol/L PE (*P* < 0.05, *n* = 4) (Fig. 7C). These findings are thus in line with earlier studies that have reported basal VSM *R*_m_ values approximating 100 MΩ (Hirst et al. 1986; Yamamoto et al. 2001), along with increases in Rm following *α*_1_‐adrenoceptor activation in arterial VSM (Kajiwara et al. 1981).

### The L‐type Ca^2+^ channel blocker nifedipine impairs *α*_1_‐adrenoceptor‐mediated enhancement of endothelium‐induced vasodilation

**Figure 8:**
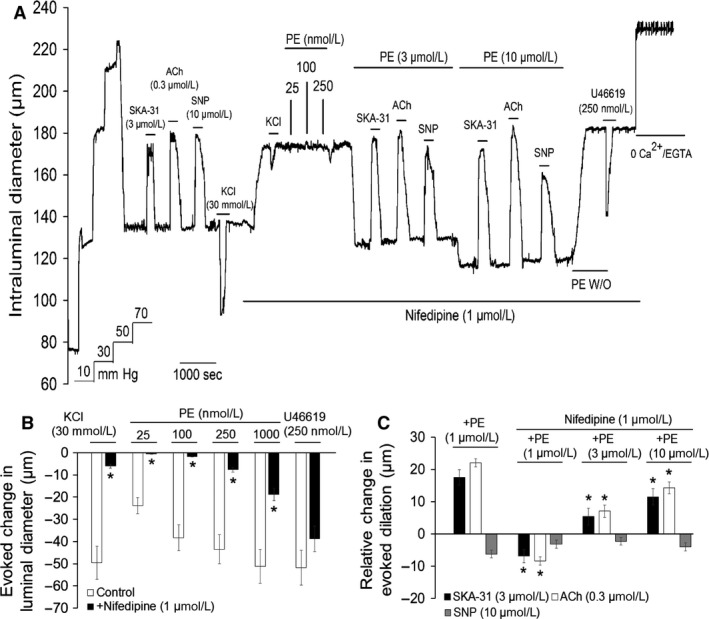
Calcium entry via nifedipine‐sensitive Ca^2+^ channels is an essential process contributing to PE‐induced augmentation of endothelium‐dependent vasodilation. The representative tracing in panel (A) displays vasodilatory responses to 0.3 *μ*mol/L ACh, 3 *μ*mol/L SKA‐31 and 10 *μ*mol/L SNP, along with a constrictor response to 30 mmol/L KCl, in a myogenic artery under control conditions, and then following bath addition of 1 *μ*mol/L nifedipine, as denoted by the horizontal bars above and below the tracing. Addition of either 3 or 10 *μ*mol/L PE in the continued presence of nifedipine produced significant arterial contraction and reduction in intraluminal diameter. Vasodilatory responses to ACh, SKA‐31 and SNP were then repeated in the presence of nifedipine + PE, followed by washout (W/O) of PE from the bath. The constrictor response to U46619 was then examined in the continued presence of nifedipine. The histogram in panel (B) quantifies the magnitude of vasoconstriction induced on top of existing myogenic tone by either 30 mmol/L KCl, PE (25–1000 nmol/L) or 250 nmol/L U46619 in the absence (control) or presence of 1 *μ*mol/L nifedipine. Data are presented as means ± SEM for 6–7 individual arteries. The asterisks (*) indicate a statistically significant difference compared with the control response, as determined by a Student's *t*‐test; *P* < 0.05. Panel (C) quantifies the relative change in magnitude of vasodilatory response observed for ACh, SKA‐31 and SNP in the presence of the indicated concentrations of PE + 1 *μ*mol/L nifedipine, relative to control responses in the absence of PE + nifedipine. For reference purposes, the bars on the left‐hand side depict the relative changes in endothelium‐dependent dilatory responses observed in the presence of 1 *μ*mol/L PE alone. The * indicates that a given value is statistically different from zero, as determined by a Student's *t*‐test.

Activation of *α*_1_‐adrenoceptors in VSM leads to elevation of cytosolic Ca^2+^ via intracellular release and entry via voltage‐gated, L‐type Ca^2+^ channels, and it has been speculated that this elevated Ca^2+^ may contribute to PE‐mediated enhancement of endothelium‐dependent vasodilation (Dora et al. 1997; Yashiro and Duling 2000). To investigate the potential contribution of L‐type Ca^2+^ channel activity to this phenomenon, we treated myogenically active, cremaster arteries with nifedipine, an established blocker of L‐type Ca^2+^ channels, to selectively interfere with Ca^2+^ signaling in the arterial smooth muscle. Note that vascular endothelial cells do not express L‐type Ca^2+^ channels (Colden‐Stanfied et al. 1987; Adams et al. 1989; Garland et al. 2017). As shown by the representative tracing in Figure 8A, myogenically active cremaster arteries exhibited typical dilatory responses to ACh, SKA‐31, and SNP, along with a robust and reversible constriction to bath‐applied 30 mmol/L KCl (N.B. NaCl in the Krebs’ buffer was replaced with KCl to maintain isotonicity). Exposure to 1 *μ*mol/L nifedipine significantly inhibited myogenic tone, but did not abolish it, in agreement with earlier results (Kotecha and Hill 2005). Nifedipine treatment, however, largely prevented the contractile response to KCl (Figs. 8A and B), demonstrating effective blockade of depolarization‐mediated Ca^2+^ entry into VSM. Nifedipine also inhibited contractile responses to PE (25 nmol/L to 1 *μ*mol/L) (Fig. 8A and 8B), in agreement with the major contribution of L‐type Ca^2+^ channels to *α*_1_‐adrenergic‐induced vasoconstriction (Miriel et al. 1999; Moosmang et al. 2003). Importantly, nifedipine treatment abolished the ability of 1 *μ*mol/L PE to augment vasodilatory responses to ACh and SKA‐31 (Fig. 8C). However, with higher levels of *α*_1_‐adrenoceptor stimulation (i.e. 3 and 10 *μ*mol/L PE), robust constrictions were observed in the presence of nifedipine, and vasodilatory responses to ACh and SKA‐31 exhibited modest, but significant augmentation, compared with control responses obtained in the absence of nifedipine + PE (Fig. 8A and C). In contrast to these observations, dilatory responses to SNP in the presence of nifedipine + PE remained similar to those observed in the presence of 1 *μ*mol/L PE alone (Fig. 8C). Note that under control conditions, we were unable to faithfully record vasoactive responses in myogenic arteries exposed to either 3 or 10 *μ*mol/L PE alone, as the powerful constrictions prevented reliable and consistent tracking of intraluminal diameter. Interestingly, nifedipine exposure did not significantly impair the constriction induced by 250 nmol/L U46619 (Fig. 8B), indicating only a limited role for L‐type Ca^2+^ channel activity in this agonist‐evoked response.

## Augmentation of ACh‐evoked dilation by *α*_1_‐adrenoceptor stimulation is blunted by inhibitors of eNOS and endothelial KCa channels

**Figure 9:**
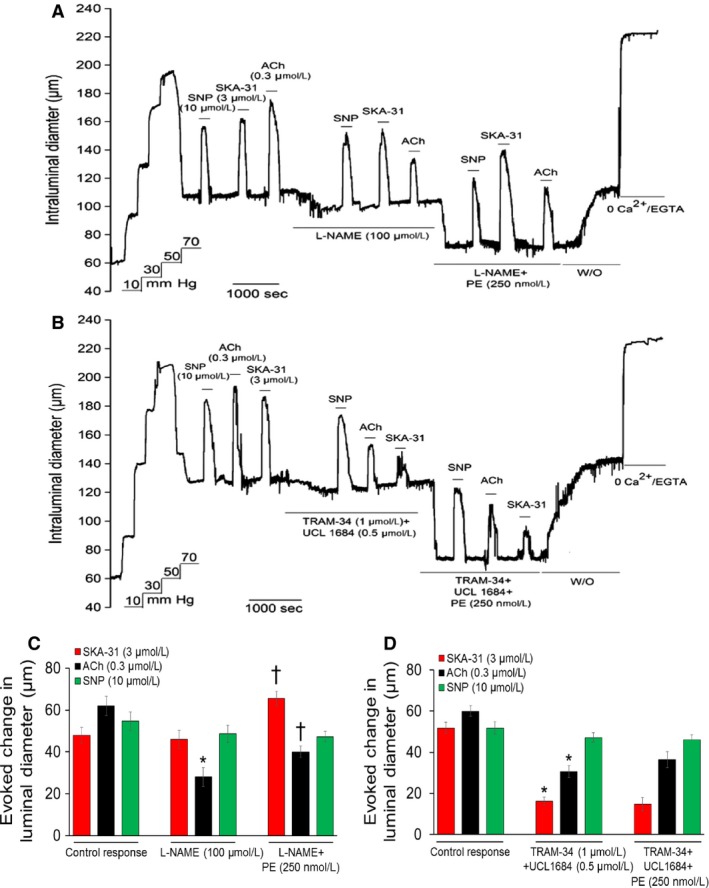
Pharmacologic inhibition of either eNOS or endothelial K_C__a_ channels blocks PE‐induced augmentation of evoked vasodilation. Panel (A) shows a representative tracing of vasodilatory responses evoked by ACh, SKA‐31 and SNP in a myogenic artery under control conditions, in the presence of the eNOS inhibitor L‐NAME (0.1 mmol/L), and then in the presence of L‐NAME + of 0.25 *μ*mol/L PE, as indicated by the horizontal bars above and below the tracing. Bath exposure to 0 Ca^2+^ Krebs’ solution + 2 mmol/L EGTA produced maximal passive diameter of the artery at 70 mmHg. In panel (B), vasodilatory responses to ACh, SKA‐31 and SNP were observed in the absence and presence of the endothelial K_C__a_ channel blockers TRAM‐34 (1 *μ*mol/L) + UCL‐1684 (0.5 *μ*mol/L), followed by TRAM‐34 + UCL‐1684 + 0.25 *μ*mol/L PE. The histograms in panels (C) and (D) quantify the absolute changes in intraluminal diameter evoked by ACh, SNP and SKA‐31 for treatments involving either L‐NAME (panel C) or TRAM‐34 + UCL‐1684 (panel D). Data are presented as means ± SEM for 3 individual arteries for each protocol. The asterisk (*) indicates a statistically significant difference compared with the control response for a given dilatory agent; the cross symbol (†) indicates a significant difference versus responses observed in the presence of either L‐NAME or TRAM‐34 + UCL1684 alone. Statistical analyses were performed using a one‐way ANOVA and Tukey's post hoc test; *P* < 0.05.

Two major Ca^2+^‐dependent signaling pathways in endothelium contributing to agonist‐evoked vasodilation include the activation of eNOS and the opening of K_Ca_ 2.3 and K_Ca_ 3.1 channels (Sheng and Braun 2007; Félétou and Vanhoutte 2009; Sheng et al. 2009). To examine if either pathway participated in the observed PE‐induced enhancement of endothelium‐dependent dilation, vasodilatory responses to ACh, SNP and SKA‐31 were first examined in the presence of either an inhibitor of eNOS (L‐NAME) or blockers of endothelial K_Ca_ channels (i.e. TRAM‐34 + UCL‐1684), and these responses were then reexamined following addition of 0.25 *μ*mol/L PE. As shown by the representative tracing in Figure 9A, exposure of a myogenic cremaster artery to L‐NAME alone produced a minor decrease in intraluminal diameter, as we have previously reported(Mishra et al. 2015), and a significant reduction in the ACh‐evoked dilation, but did not affect vasodilatory responses to either SKA‐31 or SNP. Further addition of 0.25 *μ*mol/L PE caused a robust constriction that augmented the vasodilatory response to ACh, as well as SKA‐31, whereas no change was observed for SNP (Fig. 9C). In the presence of L‐NAME, addition of 0.25 *μ*mol/L PE produced a significant enhancement of the ACh‐evoked dilation, however, the relative magnitude of this vasodilatory response ±PE (R2‐R1 response = 12.0 ± 1.6 *μ*m,) was blunted compared with the augmentation of ACh responses observed without L‐NAME treatment (16.7 ± 1.7 *μ*m, see Fig. 3). In contrast, L‐NAME treatment did not impact the calculated relative change in the vasodilatory response to SKA‐31 in the presence and absence of 0.25 *μ*mol/L PE (19.6 ± 3.1 *μ*m), which is comparable to that observed without L‐NAME co‐treatment (see Fig. 3). This latter result indicates that eNOS inhibition does not impact the PE‐induced enhancement of SKA‐31 evoked dilation. L‐NAME exposure did not alter the dilatory response to SNP (Fig. 9A, C), which was unaffected by the further addition of 0.25 *μ*mol/L PE.

Treatment of myogenic arteries with blockers of endothelial K_Ca_ 3.1 and K_Ca_ 2.3 channels (TRAM‐34 and UCL‐1684, respectively) produced a minor vasoconstriction (Mishra et al. 2015) and largely prevented the vasodilatory response elicited by the K_Ca_ channel activator SKA‐31, as one would anticipate (Fig. 9B). Further addition of 0.25 *μ*mol/L PE in the presence of the K_Ca_ channel blockers did not alter the magnitude of the SKA‐31 induced dilation (Fig. 9D), indicating an absence of PE‐induced augmentation. Exposure to TRAM‐34 + UCL‐1684 also blunted the ACh‐evoked dilation (Fig. 9B), and the modest change in response observed following addition of PE (R2–R1 response = 5.8 ± 2.4 *μ*m) was not statistically significant (Fig. 9D).

## Discussion

The intraluminal diameter of resistance arteries is a critical determinant of systemic blood pressure and blood flow distribution within tissues, and is regulated, in part, by the extent of luminal pressure‐induced myogenic tone. Sympathetic innervation and stimulation of resistance arteries can lead to further constriction, via activation of smooth muscle *α*‐adrenoceptors (Westcott and Segal 2013), whereas locally generated vasodilators (e.g., nitric oxide, endothelium‐derived hyperpolarization) oppose contractile processes. Several reports have demonstrated that *α*_1_‐adrenergic stimulation of vascular smooth muscle (VSM) can positively influence the effectiveness of endothelium‐evoked vasodilation in peripheral resistance arteries, and at a cellular level, this phenomenon is associated with a rise in endothelial free Ca^2+^ (Dora et al. 1997, 2003; Yashiro and Duling 2000; Nausch et al. 2012; Garland et al. 2017). In this study, we independently reproduce and expand these observations by describing a systematic characterization of VSM *α*_1_‐adrenoceptor‐dependent modulation of endothelium‐mediated vasodilation in isolated, myogenically active resistance arteries from rat cremaster muscle. Our findings demonstrate that the *α*_1_‐adrenoceptor‐driven augmentation of endothelium‐dependent vasodilatory responses requires a threshold concentration of *α*_1_‐adrenergic agonist, and occurs in a positive fashion suggestive of increasing *α*_1_‐adrenoceptor occupancy/activation (see Figs. 1 and 2). Physiologically, this influence of *α*_1_‐adrenoceptor activity on endothelium‐mediated vasodilation would act in a negative feedback fashion to limit *α*_1_‐adrenergic‐induced decreases in intraluminal diameter of resistance arteries that may arise with augmented levels of sympathetic nerve activity. Limiting such constriction would prevent restrictions and/or dysregulation in tissue blood flow and excessive rises in systemic blood pressure.

Experimentally, it is evident from the tracing in Figure 1 and the cumulative data in Figures 2 and 3 that the *α*_1_‐adrenergic agonist phenylephrine (PE) acts in a concentration‐dependent manner to enhance the endothelium‐dependent vasodilatory responses evoked by the Ca^2+^‐mobilizing agonist acetylcholine (ACh) and SKA‐31, a small molecule activator of endothelial Ca^2+^‐activated K^+^ channels (i.e. K_Ca_ 2.3 and K_Ca_ 3.1) (Sankaranarayanan et al. 2009). By utilizing the magnitude of PE‐induced contraction as a functional correlate of *α*_1_‐adrenoceptor activation, it is apparent from Figures 3 and 4 that a positive relation exists between the degree of *α*_1_‐adrenergic stimulation and the enhancement of endothelium‐dependent vasodilation, recapitulating the hypothesized relation presented in Figure 1B. Denuding the vascular endothelium is known to prevent this enhancement of vasodilatory responses by *α*_1_‐adrenoceptor stimulation (Dora et al. 1997; Garland et al. 2017). In contrast to ACh and SKA‐31, exposure to PE blunted the dilatory response to the nitrovasodilator sodium nitroprusside (SNP), which is known to act directly on VSM to cause relaxation via a NO/cGMP/PKG signaling cascade (Feelisch 1998). Functionally, the positive and negative actions of PE on the vasodilatory actions of ACh, SKA‐31 and SNP were mimicked by the native adrenoceptor agonist norepinephrine (1 *μ*mol/L, Figs. 3 and 4). Earlier work has demonstrated that the *α*_1_‐adrenoceptor is the predominant subtype in the rat vessels utilized in this study (i.e. cremaster 1A arteries)(Faber 1988; Ohyanagi et al. 1991; Moore et al. 2010a), and that NE‐induced vasoconstriction in these arteries is largely blocked by the *α*_1_‐adrenergic antagonist prazosin, but not by the *α*_2_‐adrenoceptor antagonist rauwolscine. In murine cremaster arteries, inhibition of *β*‐adrenoceptors with propranolol was found to have no effect on either PE or NE‐induced vasoconstriction(Moore et al. 2010a), implying that these agonists act largely via *α*‐adrenoceptors in cremaster arteries. Collectively, these observations point to a major contribution of endothelium and VSM *α*_1_‐adrenoceptors to the PE and NE‐induced enhancement of evoked vasodilation. Interestingly, our data further imply that the rise in VSM Ca^2+^ observed in myogenically active resistance arteries at physiologic pressures (e.g., 70 mmHg) (Meininger et al. 1991; Zou et al. 2000) is insufficient to promote enhancement of endothelium‐dependent vasodilation, as we observed no difference in evoked vasodilatory responses in the absence and presence of a low concentration of PE (i.e., 25 and 100 nmol/L), even though this latter condition increased contraction and is reported to elevate cytosolic‐free Ca^2+^ in cremaster VSM (Potocnik and Hill 2001). Mechanistically, Dora and colleagues have suggested that enhancement of endothelium‐dependent vasodilation via VSM signaling requires the recruitment of additional L‐type Ca^2+^ channels in VSM, as Ca^2+^ entry due solely to myogenic mechano‐transduction may be compartmentalized from the Ca^2+^ signaling underlying augmentation of endothelium‐dependent dilation (Garland et al. 2017).

In contrast to the enhancement observed with *α*_1_‐adrenergic stimulation, exposure of myogencially active arteries to the thromboxane A_2_ receptor (TP) agonist U46619 (250 nmol/L) significantly impaired dilatory responses to ACh, SKA‐31 and SNP, despite producing a degree of constriction comparable to that observed in the presence of either 1 *μ*mol/L PE or 1 *μ*mol/L NE (Figs. 4 and 6). This differential response likely reflects the prominent role played by Ca^2+^ sensitization of the VSM contractile machinery in the TP receptor‐induced vasoconstriction and less dependence on evoked Ca^2+^ dynamics (Himpens et al. 1990; Tosun et al. 1998; Wilson et al. 2005). If elevations in VSM Ca^2+^ represent a critical step in the cellular mechanism driving enhancement of endothelium‐dependent vasodilatory responses, then other stimulatory events that elevate cytosolic Ca^2+^ in VSM may augment these responses in a manner comparable to *α*_1_‐adrenoceptor activation. 4‐Aminopyridine (4‐AP) is an established blocker of voltage‐gated K^+^ channels in VSM that produces membrane depolarization and promotes contraction via the opening of L‐type Ca^2+^ channels (Knot and Nelson 1995; Nelson and Quayle 1995). Exposure of myogenically active cremaster arteries to 1 mmol/L 4‐AP produced a robust constriction comparable to those observed with PE and NE, and significantly augmented the vasodilatory response to SKA‐31. In line with observations for PE and NE, 4‐AP did not alter the dilatory response to SNP (Fig. 6). Surprisingly, 4‐AP did not significantly enhance the ACh‐induced vasodilation. This observation likely reflects the reported ability of 4‐AP (0.1–3 mmol/L) to interact directly with muscarinic receptors to impair ligand binding (Lai et al. 1985). 4‐AP binding to muscarinic receptors in the heart also activates K^+^ currents in cardiac pacemaker cells (Navarro‐Polanco and Sánchez‐Chapula 1997; Arechiga‐Figueroa et al. 2010). As a result of these 4‐AP/receptor interactions, the ability of ACh to activate endothelial muscarinic receptors was possibly compromised in the presence of 1 mmol/L 4‐AP, thereby reducing the efficacy of ACh‐mediated vasodilation (e.g., effective receptor occupancy by ACh reduced from 50 to 60% to a lower level) and preventing full expression of the *α*_1_‐adrenergic‐induced enhancement. Recently, Jepps and colleagues have reported that 4‐AP caused relaxation of NE‐contracted rat mesenteric arteries, and that this action could be prevented by blockers of Kv7.4 and BK_Ca_ currents (Khammy et al. 2018). Although 4‐AP typically produces constriction, not relaxation of myogenic arteries (e.g., see Fig. 6, Telezhkin et al. (2007) and Kang et al. (2009)), these findings suggest that 4‐AP may have additional effects that could limit the augmentation of ACh‐induced dilation. It is also noteworthy that 4‐AP treatment enhanced the vasodilation evoked by pinacidil (3 *μ*mol/L), an opener of ATP‐sensitive K^+^ channels prominently expressed in VSM. By directly activating K^+^ channels, SKA‐31 and pinacidil stimulate hyperpolarizing membrane currents in vascular endothelium and smooth muscle, respectively, which ultimately cause VSM hyperpolarization. Given this action, it is not perhaps surprising that these agents evoke greater vasodilatory responses in 4‐AP‐treated myogenic arteries that exhibit greater membrane resistance and depolarization, relative to arteries experiencing only pressure‐induced myogenic constriction.

Similar to 4‐AP, *α*_1_‐adrenergic stimulation also produced membrane depolarization in myogenically contracted cremaster arteries that was associated with a significant rise in membrane electrical resistance (Fig. 7). This observation is thus consistent with earlier reports describing the inhibition of outward K^+^ currents in vascular smooth muscle by *α*_1_‐adrenergic agonists, such as PE (Bonev and Nelson 1996; Mistry and Garland 1999; Li and Janssen 2002). As predicted by Ohm's Law (*V* = *I* × *R*), an increase in steady‐state membrane resistance would allow for a greater change in membrane potential for a given amount of current crossing the VSM membrane. Mechanistically, this observation implies that a fixed amount of hyperpolarizing current traveling from the endothelium to the adjacent smooth muscle would generate an increasingly larger hyperpolarization in VSM as membrane resistivity rises. Thus, enhancement of SKA‐31‐mediated vasodilation in the presence of *α*_1_‐adrenoceptor activation may result from a modest rise in endothelial K_Ca_ channel activity, due to elevated cytosolic Ca^2+^, along with an increase in VSM membrane resistance. In combination, these two factors would lead to an augmented hyperpolarization of the VSM that would dampen voltage‐gated Ca^2+^ channel activity, Ca^2+^ entry and VSM contractility.

Voltage‐gated, L‐type Ca^2+^ channels are known to play an important role in pressure‐induced myogenic constriction and *α*_1_‐adrenoceptor‐evoked contractile responses (Davis and Hill 1999; Hill et al. 2001; Kotecha and Hill 2005), but their functional importance for the *α*_1_‐adrenergic‐mediated augmentation of endothelium‐mediated vasodilation remains poorly understood. The data in Figure 8 demonstrate that blockade of L‐type Ca^2+^ channels with the classic dihydropyridine inhibitor nifedipine (1 *μ*mol/L) effectively abolished the contractile response of myogenic arteries to 30 mmol/L KCl and strongly attenuated the constriction evoked by 1 *μ*mol/L PE; collectively, these contractility data strongly imply that nifedipine treatment prevented a rise in VSM cytosolic Ca^2+^ to support KCl and PE‐stimulated contractions. Critically, the presence of nifedipine changed the effect of 1 *μ*mol/L PE on endothelium‐mediated vasodilation from positive to negative (Fig. 8C). However, the *α*_1_‐adrenoceptor‐mediated enhancement could be functionally restored by exposing nifedipine‐treated arteries to higher concentrations of PE (i.e., 3 and 10 *μ*mol/L) that generated considerable vasoconstriction; this latter observation would be consistent with increasing levels of VSM cytosolic Ca^2+^ (Potocnik and Hill 2001; Garland et al. 2017). Mechanistically, these putative Ca^2+^ elevations could arise from internal Ca^2+^ store release and/or external Ca^2+^ entry via nifedipine‐insensitive channels or transporters. Taken together, these data indicate that L‐type Ca^2+^ channels contribute not only to the normal *α*_1_‐adrenoceptor‐induced vasoconstriction in myogenically active cremaster arteries, but also to the *α*_1_‐adrenergic‐mediated augmentation of endothelium‐dependent vasodilatory responses, which is in agreement with the findings of Dora and coworkers (Garland et al. 2017). Reduction in L‐type channel activity decreases the efficacy with which *α*_1_‐adrenoceptor stimulation triggers augmentation of endothelium‐mediated vasodilation, most likely by eliminating a major pathway for Ca^2+^ elevation in VSM.

Mechanistically, augmentation of endothelial Ca^2+^ signaling in the presence of PE could impact the activation of both eNOS and endothelial K_Ca_ channels by Ca^2+^‐mobilizing vasodilators (e.g., ACh). Selective inhibition of eNOS activity with L‐NAME significantly decreased the PE‐induced enhancement of ACh‐evoked vasodilation, and a similar effect was observed in the presence of the K_Ca_ channel blockers TRAM‐34 + UCL‐1684 (Fig. 9). These data strongly suggest that the activities of both eNOS and endothelial K_Ca_ 2.3 and K_Ca_ 3.1 channels participate in the observed augmentation of agonist‐evoked dilation in the presence of PE, which would agree with their known Ca^2+^ sensitivity. At the cellular level, we have observed that endothelial K_Ca_ channel activity is important for agonist‐evoked NO synthesis (Sheng and Braun 2007) and augmentation of K_Ca_ 2.3 and K_Ca_ 3.1 currents promotes greater endothelial hyperpolarization (Sheng et al. 2009). Similarly, Dora and colleagues have reported that pharmacologic inhibition of K_Ca_ 2.3 and K_Ca_ 3.1 channels interfered with the PE‐induced augmentation of endothelium‐dependent regulation of myogenic tone in cremaster arteries (Garland et al. 2017).

**Figure 10:**
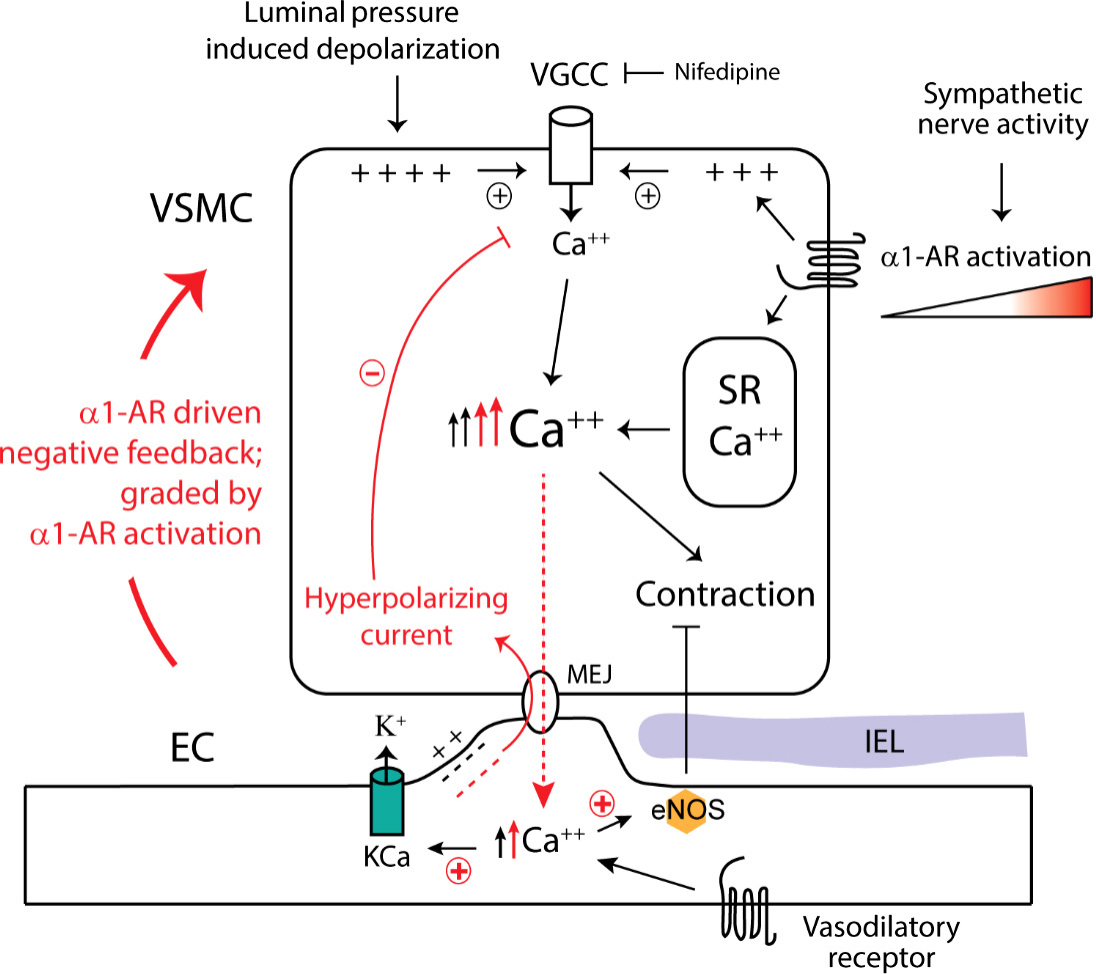
Cartoon summarizing the putative cellular processes contributing to *α*_1_‐adrenoceptor‐mediated enhancement of endothelium‐dependent vasodilation in skeletal muscle resistance arteries. Elevation of intraluminal pressure promotes vascular smooth muscle cell (VSMC) depolarization, activation of L‐type, voltage‐gated Ca^2+^ channels (VGCCs) and Ca^2+^ entry. The rise in cytosolic‐free Ca^2+^ then drives myofilament‐based contraction and a reduction in intraluminal diameter. Release of norepinephrine from perivascular sympathetic nerves stimulates *α*_1_‐adrenoceptors (*α*1‐ARs) on the VSMC membrane, leading to IP_3_‐mediated Ca^2+^ release from the sarcoplasmic reticulum (SR) and VGCC activation via membrane depolarization, both of which augment cytosolic‐free Ca^2+^ and promote contraction. As the level of VSM cytosolic Ca^2+^ rises with increasing *α*_1_‐adrenoceptor stimulation and passes a critical threshold, further rises in VSM Ca^2+^ lead to diffusion of cytosolic Ca^2+^ and/or IP_3_ from the VSM to the adjacent vascular endothelium via myoendothelial junctions (MEJ). Elevation of endothelial cell free Ca^2+^ augments the initial activation of eNOS and Ca^2+^‐activated K^+^ channels (K_C__a_) by vasodilatory stimuli. The enhanced opening of endothelial K_C__a_ channels generates a more robust hyperpolarizing current that travels back to the VSM via MEJs to cause VSM hyperpolarization, a reduction in VGCC activity and cytosolic‐free Ca^2+^, thereby limiting the magnitude of contraction. The main cellular events underlying this *α*_1_‐adrenoceptor‐driven, negative feedback process are highlighted in red.

The schematic in Figure 10 highlights the major cellular events/pathways implicated in the *α*_1_‐adrenergic enhancement of endothelium‐mediated vasodilation in skeletal muscle resistance arteries. Mechanistically, pressure‐induced depolarization of VSM acts as the primary stimulus for elevation of cytosolic Ca^2+^ via voltage‐gated Ca^2+^ channels and the initiation of myogenic contraction (Davis and Hill 1999; Hill et al. 2001), which can be further increased by a superimposed stimulus that also elevates VSM Ca^2+^ (e.g., *α*_1_‐adrenoceptor agonist). As the secondary stimulus elevates cytosolic Ca^2+^ beyond a threshold level, or possibly in a distinct subcellular compartment, a parallel rise in endothelial Ca^2+^ is initiated, likely by the gradient‐driven diffusion of VSM Ca^2+^ and/or the transcellular movement of a Ca^2+^ mobilizing second messenger, such as IP_3_ (Isakson et al. 2007; Nausch et al. 2012), via gap junction channels located at myoendothelial connections between the two cell types (Sandow et al. 2006; Straub et al. 2014). Elevated endothelial Ca^2+^ would then enhance the actions of endothelium‐dependent, Ca^2+^ mobilizing vasodilatory stimuli (e.g. ACh) that elicit K_Ca_ 2.3 and/or K_Ca_ 3.1 channel‐mediated hyperpolarizing K^+^ currents and nitric oxide production that act on the adjacent VSM to dampen contraction. In support, other investigators have provided compelling evidence that *α*_1_‐adrenergic stimulation of skeletal muscle resistance arteries can elevate endothelial cell (EC) free Ca^2+^ and augment endothelium‐dependent vasodilation (Dora et al. 1997; Yashiro and Duling 2000; Tran et al. 2012; Garland et al. 2017). In isolated mouse mesenteric arteries, Nausch and colleagues (Nausch et al. 2012). have reported similar observations following electrical nerve stimulation or direct application of PE. Importantly, Dora and colleagues have demonstrated that elevation of endothelial cell free Ca^2+^ by PE in rat cremaster arteries is blocked in the presence of nifedipine (Garland et al. 2017). These investigators have further shown that endothelial K_Ca_ channels are important targets for PE‐induced elevations in endothelial Ca^2+^, as inhibition of these channels prevented the enhanced vasodilation observed in the presence of PE (Garland et al. 2017). This conclusion thus agrees with the observed PE‐induced augmentation of SKA‐31 mediated dilation described in our study. Elevation of VSM Ca^2+^ via *α*_1_‐adrenergic signaling is thus capable of initiating a negative feedback mechanism that recruits the adjacent endothelium and serves as a “brake” to limit the magnitude of stimulus‐evoked VSM contraction in skeletal muscle resistance arteries (highlighted by red shading in the schematic).

We speculate that the observed feedback regulation of VSM contractility by endothelium‐dependent signaling in *α*_1_‐stimulated skeletal muscle resistance arteries contributes to “functional sympatholysis”, a phenomenon in which sympathetic vasoconstriction in exercising skeletal muscle exhibits attenuation (Remensnyder et al. 1962), thereby allowing blood flow to match metabolic demand (Hansen et al. 2000). Functional sympatholysis has been described in the vasculature of rodent and human skeletal muscle (VanTeeffelen and Segal 2003; Moore et al. 2010b; Hearon and Dinenno 2016; Hearon et al. 2016), and may impact both local dilatory responses and conducted vasodilation in skeletal muscle. In addition, our data suggest that a low level of *α*_1_‐adrenoceptor activation would promote/support the elevated vascular resistance to blood flow typically observed in resting skeletal muscle (Saltin et al. 1998), which would then “flip” to enhance active vasodilation once a “threshold” level of *α*_1_‐adrenergic stimulation is attained (Fig. 2A). Finally, our findings may have important implications for the regulation of blood flow and blood pressure under conditions involving endothelial dysfunction (e.g., type 2 diabetes. ageing). Impairment of endothelial function may compromise the robustness of the observed *α*_1_‐adrenoceptor‐driven negative feedback pathway, thereby limiting the capacity of resistance arteries in exercising muscle to dilate in the face of elevated sympathetic tone. This deficit could thus compromise the pattern and/or volume of blood flow in the skeletal circulation by maintaining arteries in an overconstricted state (Saltin and Mortensen 2012; Hearon and Dinenno 2016), and possibly lead to elevated systemic blood pressure. Similar consequences would be predicted in other vascular beds (e.g., mesentery) exhibiting *α*_1_‐adrenoceptor‐mediated enhancement of endothelium‐dependent vasodilation (Nausch et al. 2012).

## Conflict of Interest

The authors declare no conflicts of interest, or competing interests in association with this study.
